# Environmental impact of orthodontic treatment: a simplified fast-track comparative life-cycle impact assessment of self-ligating metal brackets and clear aligners

**DOI:** 10.1093/ejo/cjag025

**Published:** 2026-05-12

**Authors:** Maria Johanna Jacoba Heezen, Geer M van den Dungen, Mette A R Kuijpers, Edwin M Ongkosuwito

**Affiliations:** Department of Orthodontics and Craniofacial Biology, Radboud University Medical Center, Philips van Leydenlaan 25, Nijmegen 6525 EX, The Netherlands; Orthodontie Amersfoort, Charlotte de Bourbonlaan 2, Amersfoort 3818 DJ, The Netherlands; Department of Orthodontics and Craniofacial Biology, Radboud University Medical Center, Philips van Leydenlaan 25, Nijmegen 6525 EX, The Netherlands; Amalia Cleft and Craniofacial Centre, Radboud University Medical Centre, Philips van Leydenlaan 25, Nijmegen 6525 EX, The Netherlands; Department of Orthodontics and Craniofacial Biology, Radboud University Medical Center, Philips van Leydenlaan 25, Nijmegen 6525 EX, The Netherlands; Amalia Cleft and Craniofacial Centre, Radboud University Medical Centre, Philips van Leydenlaan 25, Nijmegen 6525 EX, The Netherlands

**Keywords:** environmental impact, life-cycle assessment, carbon footprint, environment, sustainability, clear aligners, fixed appliances

## Abstract

**Objective:**

Sustainability is a prominent topic in modern-day society, with a trend toward reducing environmental impact. The growing amount of plastics and the release of greenhouse gases during waste management raise concerns. This study compares the environmental burden of clear aligners- and self-ligating metal brackets (fixed appliances) to identify the production phase where improvements could best reduce impact.

**Methods:**

A simplified fast-track life cycle impact assessment (LCIA) was performed for four illustrative patient cases (two aligner, two fixed appliances). Data were derived from the Industrial Design & Engineering Materials open access database (Idemat, 2024) and manufacturer specifications. Environmental indicators were carbon footprint (kg CO_2_-eq), cumulative energy demand [megajoules (MJ)], and total ReCiPe score [Points (Pt)].

**Results:**

Clear aligners show a higher environmental impact in all categories. Mean carbon footprint for clear aligners ranged from 20.58 to 24.75 kg CO_2_ and 0.22 kg CO_2_ in fixed appliance treatment. The Total ReCiPe ranges 0.54–0.66 Pt) for clear aligners compared with 0.01 Pt in treatment with self-ligating metal brackets. These results should be interpreted cautiously, as they may overestimate true magnitudes.

**Limitations:**

This study used a simplified LCIA, incorporating assumptions substantiated by research whenever possible.

**Conclusions:**

Within the limitations of a small sample size and a simplified model, fixed appliance treatment showed a lower environmental impact than clear aligners. The results are exploratory and require validation through larger, clinically based studies incorporating sensitivity analyses and multi-source datasets. The “materials” phase seems a key contributor, stimulating environmentally conscious decision making.

## Introduction

Sustainability is among the most prominent topics in contemporary society, with the primary objective of minimizing waste production to the greatest extent possible. Orthodontic treatment begins with an anamnesis and record taking leading to the development of a treatment plan, during which various options are discussed and presented to the patient. When the treatment plan leads to the options fixed appliances or clear aligners, the final decision is typically based on factors such as efficiency; effectiveness; aesthetics; patient and orthodontist preferences; and treatment costs. When the environmental impact of different treatment options is added to these factors, this may lead to an alternative choice that may be more sustainable.

The global clear aligner market size was foreshadowed to increase from USD 3.1 billion in 2021 to USD 6.1 billion in 2027 with a compound annual growth rate of 13% [1]. Currently the designated materials used for aligner fabrication are thermoplastic polymers (oil-derived), whom are non-biodegradable and when burned cause environmental pollution [2]. However, they demonstrate the essential thermophysical properties: sufficient flexibility to allow for insertion and removal, coupled with adequate rigidity to apply forces required for orthodontic tooth movement. To gain a better insight in why clear aligners are considered a burden for the environment it is of great importance to understand the current most commonly used way of manufacturing the clear aligners in contrast to the manufacturing of fixed appliances. The design of the clear aligners starts with a set-up of a digital 3D treatment plan. After several reviews and adjustments, the final treatment plan is translated to an STL file that can be transferred to a 3D printing machine. 3D models are printed and each separate clear aligner is then thermoformed; vacuum pressed on the cast; laser marked; trimmed and polished. Finally, the aligners are packed in individual plastic wrapping and shipped into customized boxes [3]. Self-ligating metal brackets are currently manufactured using the metal injection molding technique, a metal working process in which a binder material is mixed with finely-powdered metal. The mixture is then heated, shaped and solidified through injection molding [4]. After molding the bracket is assembled and the self-ligating metal plate is incorporated before the entire bracket system is polished and grinded to achieve the desired smoothness. This study specifically focuses on comparing the direct bonding of self-ligating metal brackets to clear aligners.

Life cycle analysis (LCA) assesses the environmental impact of a process from cradle-to-grave, identifying high-impact phases for optimization. Life cycle impact assessment (LCIA) translates emissions and resource use into key environmental impact scores [5]. The ReCiPe model simplifies life cycle inventory results into 18 midpoint indicators (e.g. climate change, water use) and three endpoint indicators (damage to human health, ecosystems, and resources) [6].

Although only 0.1% of the carbon emissions of a dental practice comes from waste, practices produce significant amounts of waste, including recycling, inadequate personal hygiene, clinical, hazardous and food waste. The authors strive toward a reduction in the generation of waste and appropriate management and categorization, while at the same time raising awareness of the financial and sustainable benefits [7]. Duane *et al*. published a series of seven papers on this subject, this emphasizes the importance of the execution of more research also in the field of orthodontics.

A systematic review by Robinson *et al*. [8] highlights the high carbon footprint of surgical procedures, such 76.9 kg CO_2_ for atrial fibrillation catheter ablations and 505.1 kg CO_2_ for CABG (Coronary Artery Bypass Grafting). In contrast, dental treatments like resin-based restorations emit just 0.12 kg CO_2_-eq [9], while in orthodontics, only retainers (Hawley and Essix) have been assessed, revealing a clear research gap.

The aim of this study is to bring about an estimate overview of the environmental impact of self-ligating metal brackets treatment compared with clear aligner treatment and specification of the different factors responsible by describing fast-track LCIA methods and apply these to self-ligating metal brackets and a clear aligner system within two different cases (extraction- and non-extraction case).

## Materials and methods

### Functional unit

Environmental impacts of this study are evaluated per full course of treatment. To equate the clear aligner treatment with the self-ligating metal brackets treatment, it is essential to determine the opportune functional unit. The self-ligating metal brackets could be subject to a certain amount of recycling and thus more patients might be orthodontically treated with one set of self-ligating metal brackets compared with the single person treated by a single set of clear aligners. The environmental impact of the orthodontic treatment with self-ligating metal brackets should, therefore, be divided over the amount of patients treated with clear aligners over the amount of the self-ligating metal brackets’ lifetime.

In this case study it is assumed that on average one patient is treated per set of individualized aligners which are not recycled due to them being considered medical waste (present a high infection-dissemination risk). Based on a study by Macrì *et al*. [10] the weight of each pair of aligners with their bag is ∼4.3 g; on average, a treatment is carried out with around 30–40 aligners, and considering only treatments performed up to 2020, ∼1875 tons of plastics were produced, of which the majority is medical waste with an infectious risk.

While usually metal brackets can be recycled up to ∼20 times (stated by Orthocycle), the reconditioning process can only proceed if the mechanical properties are unaffected by damages during wear or during the debonding process. However, the self-ligating brackets can be distorted (by the clinician) in various ways. Also a study by Neto *et al*. [11] observed a discreet bacterial colonization on the hooks of the brackets and the greatest formation of bacterial colonies occurred in the slot region. Therefore with a single set of self-ligating brackets we assume just one patient can be treated. Thus, in order to take into account the lifetime of the self-ligating metal brackets the functional unit in this study is the treatment of one clear aligner patient compared with the treatment of one self-ligating metal brackets case.

### Cases

**Figure 1:**
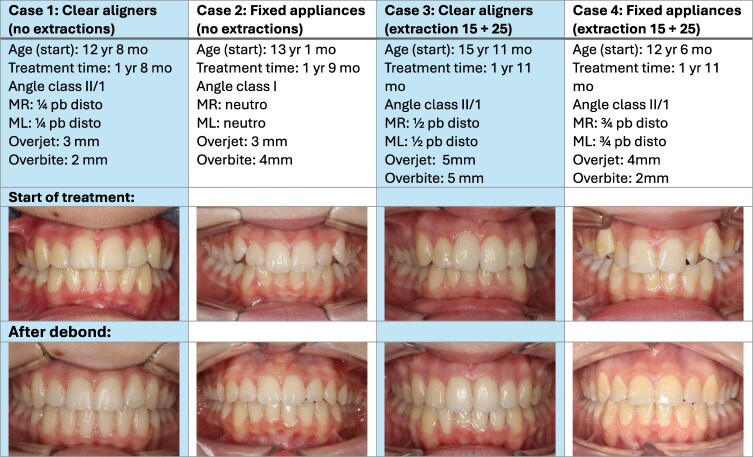
Case selection.

A total of four cases were selected (Fig. 1): two cases without extractions and two cases with extractions of the second premolars in the upper jaw—one per appliance system. The cases were selected on their similarities in occlusion and extraction protocols to allow for optimal comparison between the two treatment systems in different treatment protocols. Both treatments followed the same phased approach: record taking, treatment planning, active treatment and a retention phase. The focus of this research is the active treatment phase. All cases were selected from the patient records of the orthodontic practice “Orthodontie Amersfoort.” The reason for choosing this practice was the extensive experience on both systems by the orthodontist. All used patients or care givers gave their informed consent to be used in the study. The study protocol received approval on 4 June 2025 for not needing further approval from the medical ethics commission of the Radboud University Medical Center (Commissie Mens gebonden Onderzoek 2025-18199).

### System boundaries

**Figure 2:**
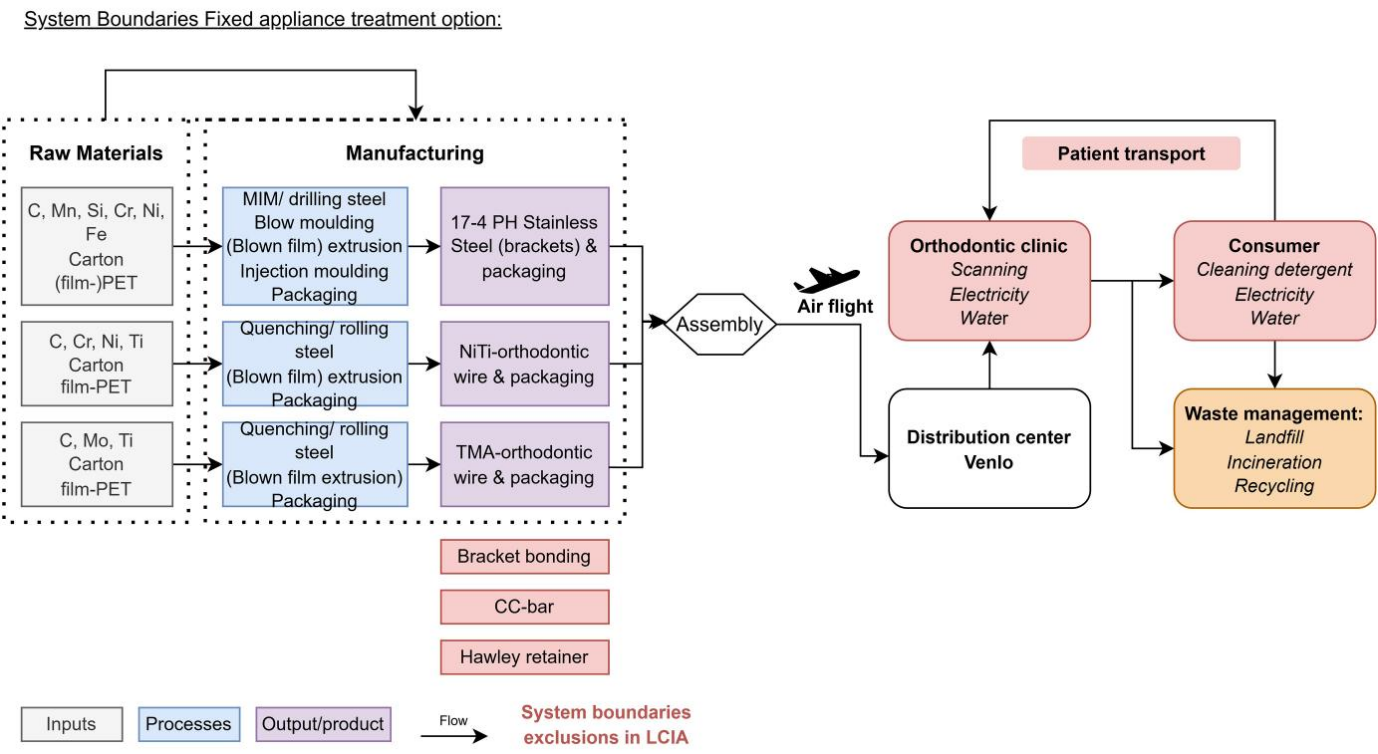
System boundaries fixed appliance treatment.

**Figure 3:**
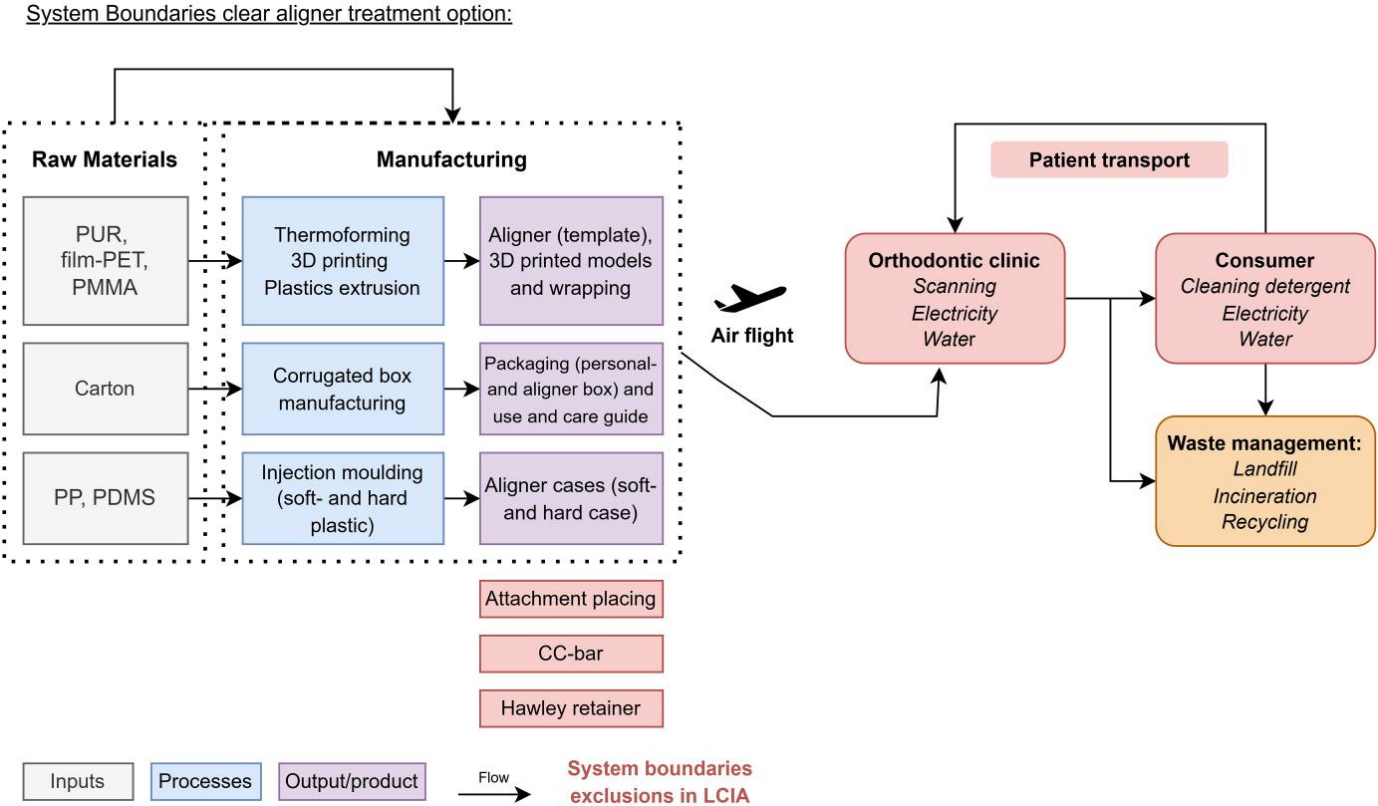
System boundaries clear aligner treatment.

The amount of materials including the material composition of the components used for the different orthodontic treatments were determined by the manufacturer’s specifications. The entire life cycle of the materials was taken into account, apart from the raw material collection, and any missing data regarding material composition that could not be traced back to the original sources was replaced with appropriate approximations based on estimates from comparable products (Supplementary Table S1: assumptions). The common steps in the fixation of orthodontic appliances (fixed brackets or aligner attachments) were assumed to be equivalent in material usage and neglected. Furthermore, both treatment systems used identical retention protocols and are therefore considered irrelevant for further analysis. Patient-related behaviors such as travel to appointments are left out of the equation since the type and distance of transport can only be roughly estimated and is difficult to quantify without specific locations and type of transport. However patients came from the same orthodontic practice area. In addition, disposal of auxiliary materials are excluded as a result of the retrospective aspect of the study in which the cases where already fully treated and specifics of auxiliary materials (e.g. elastics, ligatures, springs, buccal tubes, lock pins, bi-level pins, chains) were not written down consistently for all appointments in all cases. On top of this, the number of elastics used by patients is not quantifiable in this study design. The system boundaries are summarized in Figs. 2 and 3. The environmental indicators/comparative metrics in this study used to assess differences in impact are: carbon footprint expressed per kg CO_2_ equivalent; cumulative energy demand (CED) in megajoule (MJ); and ReCiPe 2016 endpoint World (2010) H/A expressed in Point (Pt).

### Impact categories

The treatment options are divided into three different impact categories further elaborated on below:

- The carbon footprint (per kg CO_2_ eq)—a measure of the total amount of greenhouse gases emitted directly and indirectly by one or the other treatment option. Subdivided by the different life-cycle stages (materials, production, transport and end-of-life). The carbon footprint is expressed in a carbon-dioxide equivalent to account for the varying warming potentials of different gases including but not limited to: carbon dioxide (CO_2_); methane (CH_4_); Nitrous oxide (N_2_O) and fluorinated gases.
- The CED (MJ)—measures the total energy consumption throughout the life-cycle (e.g. fossil fuels, wind, solar). Indicates the total energy impact of a treatment option.
- ReCiPe 2016 endpoint (Pt) World (2010) H/A—widely used method for evaluating environmental impact of processes or products. It combines midpoint and endpoint indicators for assessing environmental impact. Pt (=point) equals to a unit that expresses the final aggregated damage across several impact categories like human health (air quality, toxicity, etc.).

### Assumptions

A comprehensive list of assumptions applied in this study is outlined in Supplementary Table S1.

#### Raw materials

Nor the mining/retrieving of the elements of the materials (raw materials), neither the transport/packaging are taken into account. The usage of multiple materials used in the fixed appliance treatment can be deducted to their components (brackets/wires/etc.). The majority of the weight needed to determine the impact of the materials in the different life-cycle stages was determined by: Eegema BV, a specialized weighing calibration company located in Zevenbergen, the Netherlands. Some additional materials (Spark aligner use and care guide and hard- and soft aligner case) were weighed by an electronic weight scale (Mettler Toledo XSE 204). All were measured to the nearest 0.01 mg.

#### Manufacturing and packaging

The manufacturing processes of the different materials was defined by online research on the most common methods used. The final packaging was determined based on the packages received by multiple patients that had started one or the other clear aligner treatment. Machine maintenance or repairs were excluded. Instruction manuals in the packaging were included where applicable. The number of products per box were either estimated based on volumetric calculations (orthodontic wires and brackets) or the information provided by the manufacturer (maximum amount of aligners per box and the additional box for the aligner cases).

#### Transport

According to information provided by the manufacturer, the aligners were manufactured in three different countries: Mexico (Mexicali), Czech Republic (Dvur Kralove) or China (Suzhou). For the sake of the most realistic outcome the closest destination was considered the starting point, Czech Republic (Dvur Kralove). The aligners were then directly transported to the orthodontic practice, without stopping at a central distribution center. The Damon brackets/wires were produced in Mexico and the European distribution was through their distribution center in Venlo before being transported to the orthodontic practices. The manufacturer (Ormco Europe BV) states that almost all of the transport is conducted through air flight, therefore distances not traveled through air are not taken into account since these are difficult to quantify. The estimated impact of transport is depended on whether impact per weight or impact per volume is applicable, this is measured by use of the practical LCA guide from Vogtländer [12].

#### Consumer use and disposal

Usage of water or detergent agents to clean the treatment materials are negligible since teeth are assumed to be already brushed on a regular basis, which immediately cleans the wires- and brackets. The slight amount of extra time used because of the additional appliances fixed intra-orally is negligible. Even so the aligners are interchanged on a weekly basis and are therefore not assumed to be cleaned extra significantly.

## Data collection and analysis

The data in this research paper is based on desk research and the public databases (Idemat 2024) available that provide the information needed to execute a fast-track LCIA. The material data of the Damon Q was provided by Ormco Europe BV, as well as the material composition of the wires. The material data of the Spark clear aligners are patented and cannot be disclosed. It is assumed that the aligner material is made of a similar material as is used by Invisalign: multi-layer aromatic TPU from methylene diphenyl diisocyanate and 1,6-hexanediol plus additives [1].

The total mass of materials considered per case is: 5.01 kg (Case 1); 0.26 kg (Case 2); 5.91 kg (Case 3) and 0.257 kg (Case 4). The difference in mass is mostly influenced and explanatory by the amount of models that are 3D printed for each aligner treatment (3.3 kg in Case 1 and 4.1 kg in Case 3).

The fast-track life-cycle inventory was constructed by careful data extraction out of the Idemat 2024 database. The orthodontic treatment materials needed for each individual treatment option that were used for this fast-track life-cycle assessment are specified by the manufacturer. The interpretation of the results was not subjected to further statistical analysis given the substantial disparity between the results, which rendered additional quantitative comparison redundant. All data was collected in Excel and transferred to the appropriate graphs and tables as visible. Due to the nature of this article being to create an impression of the difference in environmental impact only the total ReCiPe (Pt) were envisioned and were not divided into the separate mid- and endpoint indicators.

## Results

### Fast-track LCIA

**Figure 4:**
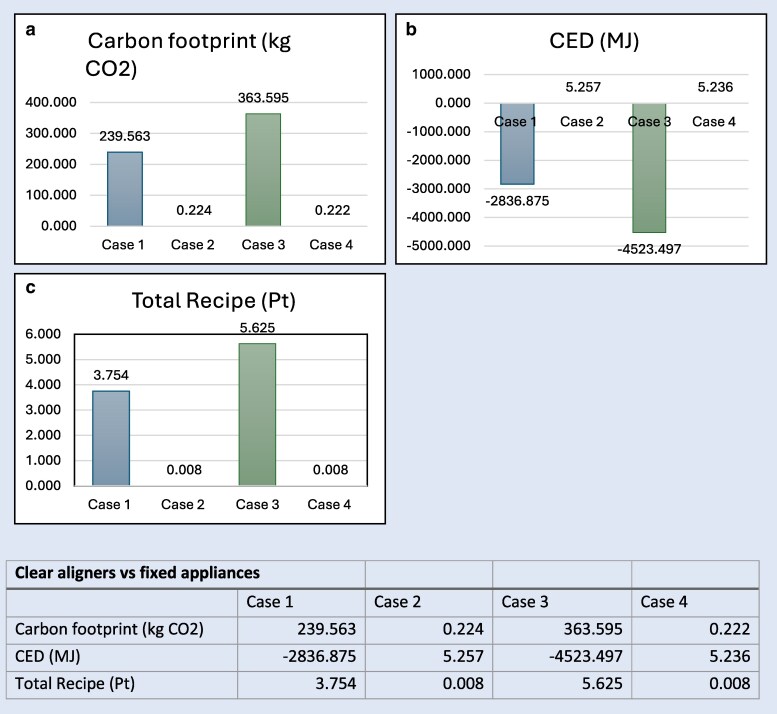
(a) Overall carbon footprint (kg CO_2_) per case. (b) Overall CED (MJ) per case. (c) Overall total ReCiPe (Pt) per case.

**Figure 5:**
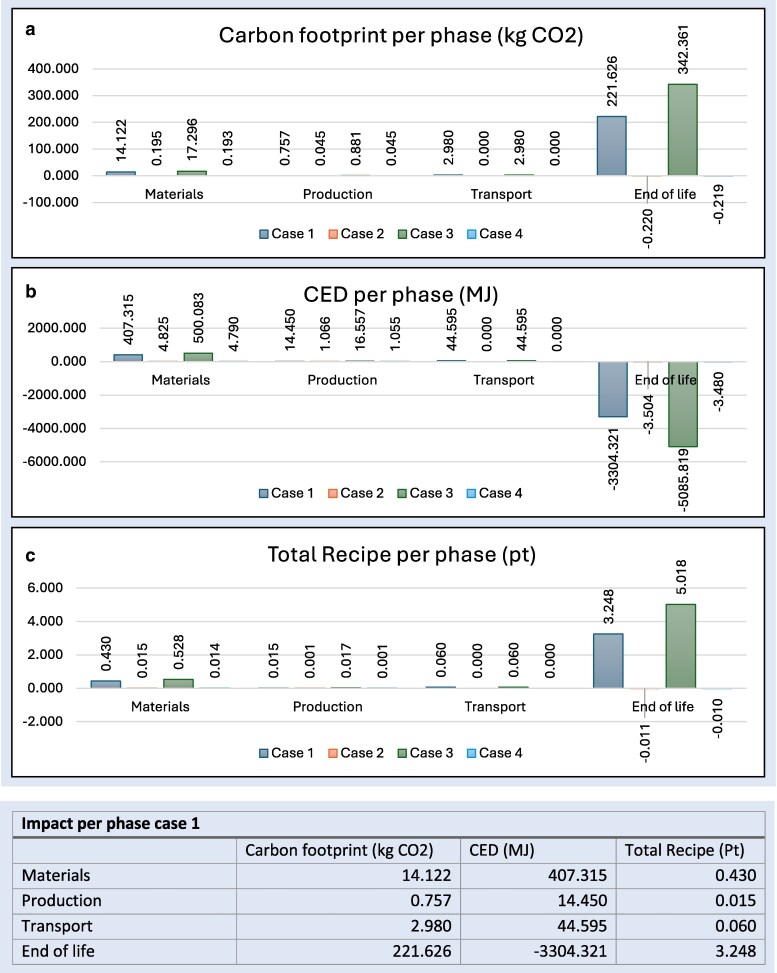

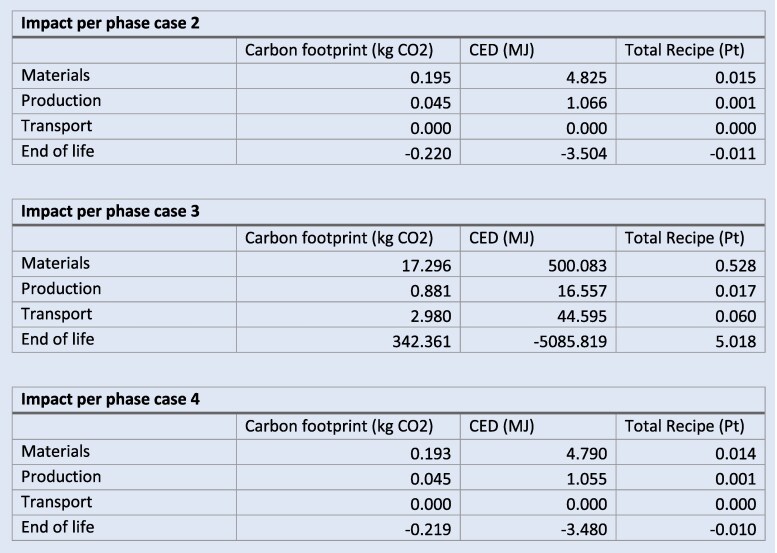
(a) Carbon footprint separated in phases per case (kg CO_2_). (b) CED per phase per case (MJ). (c) Total ReCiPe per phase per case (Pt).

The comparative fast-track LCIA data results for the differing treatment options are shown in Figs. 4 and 5. The environmental impact assessment of clear aligners (Cases 1 and 3) compared with that of fixed appliances (Cases 2 and 4) revealed substantial differences across all three environmental indicators. These being: carbon footprint (kg CO_2_); cumulative energy demand (=CED, MJ), and total ReCiPe (Pt).

Carbon footprint (overall)—Fig. 4a illustrates a distinct comparison between Cases 1 and 3 compared with Cases 2 and 4. The overall carbon footprint in clear aligner Cases 1 and 3 being respectively 20.576 kg CO_2_ and 24.749 kg CO_2_. Comparing this to the fixed appliance Cases 2 and 4 showcasing a far inferior amount of kg CO_2_ namely, 0.224 and 0.222 kg CO_2_. This can be translated into fixed appliances being the more sustainable choice with less estimated kg CO_2_ produced while being treated with one or the other treatment system.

Cumulative energy demand/CED (overall)—Fig. 4b showcases a positive CED in Case 1 and Case 3, 392.222 and 472.980 MJ that account for the amount of energy waste. A positive CED can also be found in Case 2, 5.257 MJ, and Case 4, 5.236 MJ. The CED (MJ) is in favor of the fixed appliances treatment and shows more energy waste in aligner treatment.

Total ReCiPe (Pt) (overall)—Fig. 4c depicts a total ReCiPe of 0.544 (Case 1) and 0.658 Pt (Case 3) as opposed to the far inferior amount of 0.008 Pt (Cases 2 and 4). Fixed appliances create a lower environmental burden. The absence of a universally accepted threshold for the total ReCiPe (Pt) challenges the interpretation of the results. Nonetheless, based on a study by Poore and Nemecek [13] a rough estimation was developed. To put this in perspective: a total ReCiPe below 0.01 Pt is considered low (good) and results of over 1.0 Pt are considered very high.

Carbon footprint (per phase)—Fig. 5a illustrates another distinct comparison between Cases 1 and 3 compared with Cases 2 and 4. It is evident that the main phase that influences the difference in environmental impact is the “materials” stage. The carbon footprint in this phase in clear aligner Cases 1 and 3 being respectively 14.122 and 17.296 kg CO_2_, covering 68.63% and 69.89% of the total carbon footprint. The second biggest phases of influence: the “transport” phase covering another 14.49% and 12.04% and the “End-of-life” phase covering 12.82% and 14.21%. The emissions produced through production do not profoundly influence the environmental impact of Cases 1 and 3. The fixed appliance Cases 2 and 4 produce the highest carbon footprint in the “materials” phase as well. This being 0.195 and 0.193 kg CO_2_, which is translated into 87.05% and 86.04% of the total carbon dioxide emissions. Indisputably these percentages are only this high due to the low carbon footprint of the fixed appliances.

Cumulative energy demand/CED (per phase)—Fig. 5b shows the CED in Cases 1 and 3 being positive in the materials, production and transport phase. The highest positive CED is found in the “materials phase” for Cases 1 and 3: 407.315 and 500.083 MJ. Nevertheless, in the end-of-life phase the CED is correspondingly, −75.224 and −89.342 MJ. When measured against the “End-of-life” CED of Cases 2 and 4, in sequence being −3.504 and −3.480 MJ the CED generated in the final phase does not compensate for the positive CED in the other phases in all cases.

Total ReCiPe (Pt) (per phase)—Fig. 5c exemplifies the total ReCiPe per phase. Similarly, the “materials” phase being most relevant, having the biggest impact on the total score over all phases. When looking at Cases 1 and 3 0.430 and 0.528 Pt in sequence. This being 79.04% and 79.94% of the total ReCiPe over all phases. Cases 2 and 4 depict a total ReCiPe per phase that is close to being negligible, being either lower than 0.1 Pt or negative when regarding the “End-of-life” phase.

## Discussion

The aim of this study was to provide insight into the environmental impact between two different orthodontic treatment methods, the treatment with fixed appliances compared with the treatment with clear aligners. Given the exploratory nature of this approach, the results should be interpreted as indicative differences rather than absolute values [14]. Within the limitations of this exploratory analysis, clear aligner treatment was associated with a higher estimated environmental burden in both the total ReCiPe, the carbon footprint as well as the CED. An interesting observation was the negative CED in the “End-of-life” phase, suggesting potential energy recovery. However, this apparent energy recovery does not imply superior sustainability. This discrepancy arises due to the “avoided burden.” A principle that explains how a negative CED leads to more energy recovered at the end of the production life cycle still leads to a higher amount of carbon dioxide emissions. In the case of the clear aligners there is much waste material (for instance all the aligners) that is incinerated when managing the waste. This creates a lot of heat which is credited as energy, resulting in a negative CED. Despite this, the amount of CO_2_ emissions is independent of the amount of CED. Even when a low CED exists the environmental impact can be high due to a significant amount of climate related emissions released in the atmosphere during processes like incineration of all the clear aligners that are thrown out after use, explaining the coexistence of negative CED and elevated CO_2_ outputs.

As described in a study by Peter *et al*., clear aligners are composed of thermoplastic polymers, which may persist in the environment for extended periods depending on polymer composition and environmental conditions. In addition, the incineration of the same plastics can release toxic fumes (e.g. polychlorinated biphenyls, dioxins, etc.) into the atmosphere [15]. This is confirmed by other studies stating the need for increased awareness of the consequences of NMP’s (nano- and microplastics) in the human body that may be produced by the fabrication and application of aligners, as well as the need for awareness of the environmental impact of failed prints and post-processing waste [10]. Therefore, while energy recovery processes can superficially improve CED values, they do not offset the overall environmental burden associated with aligner waste management.

Methodological limitations in the study warrant caution. First of all, the sample size (*n* = 4) is insufficient for generalization and must be seen as illustrative rather than a representative sample. Second, the use of Idemat 2024, while robust for industrial materials, is not optimized to accurately represent the proprietary medical-grade materials used in orthodontic treatment (e.g. NiTi-alloys, TMA-alloys). Entailing that the results are subjected to and influenced by the use of generic analogues as substitution for proprietary materials not available in the Idemat database (Supplementary Table S1). This is also the case for the estimated aligner material (multi-layered TPU), since there is a still active patent on the used aligner material in our cases the exact composition is unknown and the Idemat database does not support information on multi-layered TPU, meaning the closest resembling material available has been used for the data analysis [PUR (polyurethane) chemical upcycled, Supplementary Table S1]. All conversion factors used in the calculations are elucidated in Supplementary Table S1 to ensure transparency and traceability of the dataset, no additional scaling beyond standard database implementation was performed. Third, it should be taken into account that several impactful stages (raw material extraction, clinical energy use and chair time, patient travel) were excluded since they could not reliably be quantified. Lastly, the current results are highly sensitive to several key assumptions, as specified in Supplementary Table S1, emphasizing the need for a sensitivity or uncertainty analysis to quantify the eligibility of the findings.

In the orthodontic field of work only very few LCIA’s have been executed and none compared fixed appliances to clear aligners. Most of the research articles that compare two materials, methods or treatments focus on the patients and their opinion. For instance, the study by Jaber *et al*. [16] compares the effect of treatment of clear aligners versus fixed appliances by oral health-related quality of life (OHRQoL) in patients with severe crowding. They conclude that the patients’ preference is clear aligner treatment based on the OHRQoL. A study by Da Tan *et al*. [14] is one of the few that do describe a comparative life-cycle assessment of Hawley and Essix retainers. They conclude that the Hawley retainer has less environmental impact compared with the Essix. Nonetheless, this study faced shortcomings regarding the absence of materials in the database they used. Based on research in medical research databases such as Pubmed and Cochrane it can be stated that comparable research, describing the difference in environmental impact of two different treatment systems, has not yet been executed.

The different scenarios analyzed in this study, namely, extraction and non-extraction cases, produced outcomes consistent with expectations. This is illustrated in Figs. 4 and 5, although both treatment systems included two distinct clinical cases, only Cases 1 and 3 both with clear aligners demonstrated marked differences in environmental impact. This variation can be attributed to the increased material demand associated with the often greater number of aligners required in the extraction scenario. A higher aligner count directly correlates with elevated material consumption, which subsequently contributes to a larger environmental burden during the end-of-life phase. In contrast, the quantity of materials used in fixed appliance treatments remains almost constant regardless of treatment duration, and does not increase in response to the extended treatment times typically associated with extraction cases.

Focusing on the current reasons to choose for clear aligners compared with fixed appliances a trend is visible that more patients are opting for aligners for improved aesthetics as well as, in specific cases, a shorter treatment time [17]. A lot of research already focused on comparing the treatment efficacy of both systems in different situations and many arrive at the conclusion that both systems have their specific indications. Therefore a personalized treatment plan should be proposed including different treatment options so that the patient understands well and is well informed to make their best decision leading to shared decision. This approach aligns with broader trends in patient-centered care, emphasizing the importance of tailoring treatment to what patients find most important. For example this is reflected in a study with orofacial cleft (OC) patients, where the value of shared decision making is highlighted as a way to provide more individualized and effective care [18]. The environmental factor also weighing in on this choice could be an improvement in sustainable treatment methods and raise awareness.

The assumptions in this research of all transport being translated to aviation emissions, based on the shortest transport routes and information provided by the manufacturer, may result in an overestimation of a higher environmental impact. This is due to part of the transport being covered by trucks (possibly with more sustainable fuel systems) [19]. Nonetheless, the primary objective for this research is to gain better insight in the differences between the two treatment approaches. Given that this assumption was applied uniformly for both systems, it is unlikely to substantially alter the relative comparison between them, although absolute values may be affected.

Furthermore, since this paper compares two different treatment methods that both use similar retention protocols these retention methods are kept out of the equation and might reduce the possibility to extrapolate the results to another treatment method with another retention method solely based on the results in this paper.

A key concern for reclamation/recycling is the potential for cross-infection dissemination, as aligners are worn within the oral cavity and consequently classified as medical waste. The absence of a circular economy [20], that focuses on the value that exists in the material cycle through re-use and recycling of materials such as plastic and prevention of their uncontrolled release into the environment, results in a far bigger amount of plastic waste than otherwise necessary. Bichu *et al*. [1] highlight concerns regarding the environmental impact of plastic waste generated by clear aligner production and call for further research into its ecological effects. They propose direct-printed clear aligners as a potential solution, where a virtual 3D model of the patient’s teeth is directly converted into the final aligners using advanced 3D printing materials. This method could streamline the supply chain, reduce costs, and decrease environmental impact. However, current 3D printing technology has yet to reach the necessary level of advancement to fully implement this approach. Macri *et al*. [10] emphasize to recognize the fixed therapy to be an effective alternative when aligners may extend treatment times excessively. Aligners may not track due to inconsistent wearing or dental movements that are not as predictable as expected. This leads to more aligners produced than used, resulting in wasted resources and energy. Reducing this overproduction does not seem to be distributors priority. They are hoping for the introduction of campaigns promoting correct disposal and recycling techniques, with collection containers placed in flagship locations. The waste collected can be used for recycling if allowed or be utilized as a source of fuel for heat or electricity generation contributing to a more sustainable future. The need for recyclable polymers and more sustainable production methods is emphasized in a study by Peluso *et al*. [21] stating “Although stainless steel production generates higher CO_2_ emissions, it offers better recyclability. In contrast, clear aligner materials production requires more energy and generates a larger amount of nonrecyclable plastic waste.”

The use of biodegradable plastics provides society with novel end-of-life management options; however, it does not offer a sustainable solution to prevent the continuation of the majority of materials being disposed. Similarly, the trend of increasing the frequency of clear aligner changes—ranging from bi-weekly to weekly, or even alternating daily—aimed at accelerating treatment duration, contributes to a significant rise in plastic waste due to the higher consumption of aligners [15].

Future research should focus on improving and expanding life-cycle assessments within orthodontics. Prospective studies incorporating larger (clinical) datasets, sensitivity or uncertainty analyses, and multiple environmental databases (e.g. Ecoinvent) are needed to refine current estimates and strengthen the reliability of the findings. Research should also focus on incorporating comprehensive LCA frameworks and improving on the methodological limitations. In addition, expanding the system boundaries to include more clinical variables such as number of visits, auxiliary material use, clinical energy consumption, and patient travel, may improve the external validity of environmental impact assessments. Research should prioritize strategies aimed at reducing the frequency of aligner changes and promoting circular economy principles to minimize the environmental impact. Manufacturers should implement safe disposal policies for used aligners, ensuring they can be properly disinfected and recycled, while optimizing the “4R principle” (reduce, reuse, recycle, and recover). Additionally, the growing popularity of “do-it-yourself (DIY)” aligner orthodontic companies, for instance in the UK [22], presents a further challenge, as these treatments often involve the use of multiple aligners without professional oversight, leading to compromised treatment outcomes and an increase in plastic waste that is difficult to manage. The development of recyclable or biodegradable polymers and more environmentally friendly materials for 3D printing may provide new opportunities to reduce plastic waste associated with aligner production. Furthermore, research should study patient awareness and preferences in treatment choice, as well as compare the environmental impact of different orthodontic treatment methods, brands, and materials. Such research may help guide the development of more sustainable orthodontic strategies in the future.

## Conclusion

To summarize, within the limitations of four illustrative patients and the preliminary nature of a fast-track LCA, fixed appliance therapy exhibited a lower relative environmental impact than clear aligner treatment. In addition it is discovered that the “materials” phase has the biggest impact on this conclusion. These findings may contribute to increasing awareness for both patients as well as practitioners and provides a preliminary foundation for more comprehensive, clinically validated life-cycle studies aimed at reducing the ecological footprint of orthodontic care.

## Supplementary Material

cjag025_Supplementary_Data

## Data Availability

All the data that sums up the contents of this article are incorporated in the supplementary materials or are easily found elsewhere.

## References

[1] Bichu YM, Alwafi A, Liu X et al Advances in orthodontic clear aligner materials. Bioact Mater 2023;22:384–403. 10.1016/j.bioactmat.2022.10.006 36311049 PMC9588987

[2] Freitas MPM . Aligners, environmental contamination, and the role of orthodontics. Angle Orthod 2022;92:148–9. 10.2319/1945-7103-92.1.148 34929037 PMC8691470

[3] EON Dental . https://www.eondental.com/blog/how-are-clear-aligners-manufactured-a-step-by-step-guide (12 August 2024, date last accessed).

[4] Heaney D . Handbook of Metal Injection Moulding. UK, Cambridge: Woodhead Publishing, 2012.

[5] Steinmann ZJN, Huijbregts MAJ, Elshout PMF et al ReCiPe 2016: A harmonized life cycle impact assessment method at midpoint and endpoint level Report I: Characterization. RIVM, 2016. https://www.rivm.nl/bibliotheek/rapporten/2016-0104.pdf (18 September 2025, date last accessed).

[6] RIVM . LCIA: the ReCiPe model. https://www.rivm.nl/en/life-cycle-assessment-lca/recipe (29 October 2024, date last accessed).

[7] Duane RD, Harford S, Steinbach I et al Environmental sustainability and waste within the dental practice. Br Dent J 2019;226:611–8. 10.1038/s41415-019-0194-x 31028331

[8] Robinson PN, Surendran K, Lim SJ et al The carbon footprint of surgical operations: a systematic review update. Ann R Coll Surg Engl 2023;105:692–708. 10.1308/rcsann.2023.0057 37906978 PMC10626532

[9] Smith L, Ali M, Agrissais M et al A comparative life cycle assessment of dental restorative materials. Dent Mater 2023;39:13–24. 10.1016/j.dental.2022.11.007 36428112

[10] Macri M, D'Albis V, Marciani R et al Towards sustainable orthodontics: environmental implications and strategies for clear aligner therapy. Materials (Basel) 2024;17:4171. 10.3390/ma17174171 39274561 PMC11395928

[11] Neto JC, Copello FM, Bolognese AM et al Bacterial and fungal colonization on metallic and ceramic orthodontic brackets: a scanning electronic microscopy study. Int J Adv Eng Res Sci 2021;8:090–101. 10.22161/ijaers.811.9

[12] Vogtländer JG . LCA, a Practical Guide for Students Designers and Business Managers. Oegstgeest: VSSD Publishers, 2023.

[13] Poore J, Nemecek T. Reducing food's environmental impacts through producers and consumers. Science 2018;360:987–92. 10.1126/science.aaq0216 29853680

[14] Da Tan TY, Duane B, Hussein A et al Environmental sustainability of post-orthodontic dental retainers: a comparative life-cycle assessment of Hawley and Essix retainers. Eur J Orthod 2024;46:cjae012. 10.1093/ejo/cjae012 38488436 PMC10941639

[15] Peter E, J M, Ani George S. Are clear aligners environment friendly? Am J Orthod Dentofacial Orthop 2022;161:619–20. 10.1016/j.ajodo.2021.12.012 35016811

[16] Jaber ST, Hajeer MY, Burhan AS et al The effect of treatment with clear aligners versus fixed appliances on oral health-related quality of life in patients with severe crowding: a one-year follow-up randomized controlled clinical trial. Cureus 2022;14:e25472. 10.7759/cureus.25472 35663697 PMC9156343

[17] Ke Y, Zhu Y, Zhu M. A comparison of treatment effectiveness between clear aligner and fixed appliance therapies. BMC Oral Health 2019;19:24. 10.1186/s12903-018-0695-z 30674307 PMC6343314

[18] Ongkosuwito EM, Kuijpers MAR. How PRO's can contribute to what matters most to patients with orofacial clefts. J Evid Based Dent Pract 2023;23:101792. 10.1016/j.jebdp.2022.101792 36707166

[19] Sher F, Raore D, Klemeš JJ et al Unprecedented impacts of aviation emissions on global environmental and climate change scenario. Curr Pollut Rep 2021;7:549–64. 10.1007/s40726-021-00206-3 34777950 PMC8578007

[20] Narancic T, O'Connor KE. Plastic waste as a global challenge: are biodegradable plastics the answer to the plastic waste problem? Microbiology (Reading) 2019;165:129–37. 10.1099/mic.0.000749 30497540

[21] Peluso A, Murmura G, Sinjari B et al Environmental impact of orthodontics: a literature review of traditional multibracket appliances and clear aligners. Int J Dent 2026;2026:2304712. 10.1155/ijod/2304712 41522266 PMC12784171

[22] Carter A, Stokes S. Availability of ‘do-it-yourself’ orthodontics in the United Kingdom. J Orthod 2022;49:83–8. 10.1177/14653125211021607 34096369 PMC8915217

