# Supplementary material for: Environmental impact of orthodontic treatment: a simplified fast-track comparative life-cycle impact assessment of self-ligating metal brackets and clear aligners
Source: Eur J Orthod. 2026 May 12;48(3):cjag025. doi: 10.1093/ejo/cjag025 (PMC13160670; doi:10.1093/ejo/cjag025)
Supplement: cjag025_Supplementary_Data [file cjag025_supplementary_data.docx]

Supplementary Table 1: Assumptions list

| Common steps in the fixation of the orthodontic appliances | - Both treatment systems are bonded using an etching agent, bonding material and composite. We assume that the amount of materials used for this step to be roughly equivalent in the different treatment systems (fixed appliances compared to clear aligners) and therefore these factors are left out of the analysis. |
| --- | --- |
| Retention | - All cases used CC-bar fixed retainers (upper- and lower jaw) and a Hawley retainer as the designated retention method. Due to the consistency of the retention method the impact is considered equivalent and excluded. - Manufacturing of the retention devices assumed similar in both systems. |
| Spark | - Foil layer pressed on individual carton SPARK box is <1% of total weight – excluded from analysis. - Based on the article “advances in orthodontic clear aligner materials ”we assume that the aligner material is made of a similar material as is used by Invisalign: multi-layer aromatic TPU from methylene diphenyl diisocyanate and 1,6-hexanediol plus additives. Exact composition of SPARK is patented, cannot be disclosed. Since the Invisalign material is not present in the Idemat database either PUR (Polyurethane) chemical upcycled has been used as closest resembling alternative. - A maximum of 42 aligner per Spark box; every refinement transported in own box. - Personal box incorporated as an additional box (with different dimensions). - The plastic cases are made of polypropylene and the silicon cases of silicone rubber - Metal parts and mirror parts in Spark cases ±<1% of total weight and, therefore, excluded. - Software/ hardware energy use assumed negligible; production site energy use already accounted for. |
| Treatment | - The amount of elastics and chains used in both treatment methods is equivalent or the difference/ effect is negligible. |
| Use | - No additional environmental impact is taken into account, since both treatment methods are intraoral and require minimal cleaning beyond regular dental hygiene. Furthermore, the aligners are replaced on a weekly basis. |
| Travel | - Patient travel is assumed to be similar for both systems. - Aligners (Spark): Average distance assumed to be around 980km from Dvůr Králové nad Labem (Czech Republic) to Utrecht, the most central city in the Netherlands as a base to where the aligners are shipped.   - Aligners shipped directly from Czech to practices. - Assumed that the corrugated aligner box is similar in size and weight as the box used for the fixed-appliances, since fixed appliances are often ordered once a year in bigger quantities. - Transport is primarily by air (Ormco data); other modes of transportation account for a negligible amount of the total distance and are excluded from the analysis. Both for the aligners, that are transported directly from the manufacturing location to the clinics, but also that of the Damon Q treatment system which are transported from the manufacturing location and all gathered in Venlo before being transported to the designated clinic locations.   - Spark 🡪 980km from Dvur Kralove to Utrecht (Maps distance)   - Damon Q 🡪 Mexicali (mexico) 🡪 Venlo 🡪 ±8940 km - To estimate the impact of transport using the Idemat database, we need to decide between impact per weight or impact per volume, which depends on the density or weight/volume ratio of the product (see chapter 4 of the practical LCA guide from Vogtlander [7]).   - For continental freight in a standard European truck + trailer, the breakeven point is 320 kg/m3 or 0.32 ton/m3.   - For (inter)continental air freight, the breakeven point is 167 kg/m3 or 0.167 ton/m3. |
| Manufacturing | - The carbon footprint of production sites was considered, if that was an option, including all facility-related emissions (energy for machinery, heating, cooling, lighting, internal transport and indirect employee activities). The exceptions for using machine only were when the exact data could not be found in the IDEMAT database and the closest comparable process was used for the data analysis. - PMMA (Polymethyl methacrylate): Not directly listed in Idemat 2024 for SLA/DLP 3D printing, but assumed as the base resin due to its relation to acrylic photopolymers. - Extrusion is used as an alternative in the absence of LCA data for modern 3D printing.   - Energy consumption: Both extrusion and 3D printing require heating polymers; extrusion is generally more energy efficient.   - Waste Generation: 3D printing is often more material efficient, while extrusion often requires trimming of excess material.   - Energy Use: Both are energy intensive; extrusion favors bulk manufacturing, while 3D printing minimizes waste through additive production. |
| Materials | - Damon brackets: Exact composition of the brackets is known (provided by manufacturer but unavailable in the IDEMAT 2024. Therefore, modeled as the material with the closest resemblance, stainless steel (secondary, average). - NiTi-wires: Composed of nickel-titanium, modeled as nickel (primary) due to majority content. - TMA-wires: Primarily consist of titanium, modeled as titanium (primary). - MIM: Since metal injection moulding is absent from the database, steel drilling is used instead. However, taken into account must be that this process removes material to build the desired product rather dan build it by molding. This results in more waste of material. - Wire treatment: Rolling steel is assumed to be similar to quenching of orthodontic wires, though it does not always involve quenching it is in some cases combined with controlled cooling to optimize flexibility and hardness. |
